# Subjectivity: A Case of Biological Individuation and an Adaptive Response to Informational Overflow

**DOI:** 10.3389/fpsyg.2016.01206

**Published:** 2016-08-09

**Authors:** Jakub Jonkisz

**Affiliations:** Department of Management, Institute of Sociology, University of Bielsko-BialaBielsko-Biala, Poland

**Keywords:** subjectivity, conscious experience, biological individuation, information, selection, action

## Abstract

The article presents a perspective on the scientific explanation of the subjectivity of conscious experience. It proposes plausible answers for two empirically valid questions: the ‘how’ question concerning the developmental mechanisms of subjectivity, and the ‘why’ question concerning its function. *Biological individuation*, which is acquired in several different stages, serves as a provisional description of how subjective perspectives may have evolved. To the extent that an *individuated informational space* seems the most efficient way for a given organism to select biologically valuable information, subjectivity is deemed to constitute an *adaptive response to informational overflow*. One of the possible consequences of this view is that subjectivity might be (at least functionally) dissociated from consciousness, insofar as the former primarily facilitates selection, the latter action.

## Introduction

The subjective or experiential aspect of consciousness constitutes the most perplexing, and supposedly the hardest, problem for science. There is insufficient space to analyse the complicated problem of *qualia* here (for that purpose, see, for example, Dennett, 1988; Crane, 2000) or to explain the meanings and interrelations between such concepts as *phenomenal consciousness*, *first-personal perspective*, *what-it-is-like-ness*, *for-me-ness* and the like (Nagel, 1974; Jackson, 1982; Levine, 1983, 2001; Block, 1995; Chalmers, 1995, 1996; Kriegel, 2006; Bayne, 2009). However, it seems quite reasonable to assert that it is subjectivity that is in fact crucial in all those cases^1^. How might science account for the subjectivity of conscious experiences? What is the relation between consciousness and subjectivity? Are there conscious states that are not subjective, or subjective states that are not conscious? The article presents an empirically supported hypothesis about the biological origins and function of subjectivity and gives provisional answers to these questions.

## Basic Facts and a Basic Fallacy

From the inside (or subjectively), consciousness consists of a variety of experiences: i.e., feelings, emotions, desires, sensations, perceptions, dreams, thoughts, etc. From the outside (or objectively), we can observe particular sorts of behavior and can access neurophysiological data, and (in the case of humans) may also obtain verbal reports associated with states of consciousness.

Science, we might say, is essentially objective, and conscious experience essentially subjective: that is just the way they are. Hence, “…subjective, qualitative aspects of consciousness, being private, cannot be communicated directly through a scientific theory, which by its nature is public and intersubjective. Accepting this assumption does not mean that the necessary and sufficient conditions for consciousness cannot be described. It implies only that describing them is not the same as generating and experiencing them” (Edelman and Tononi, 2000, pp. 139–140). The belief that science will ever permit us to know directly what it is like to see in color, or ‘what it is like to be a bat’ or any other being different from ourselves, is fallacious when understood literally. At the same time, though, we can ask empirically valid and productive questions about the ‘*what-it-is-like-ness*’ itself. In other words, qualitative experience is a property possessed by certain biological systems, not by any scientific claims or theories. Therefore, if proponents of the so called *knowledge argument* (formulated in many versions by, e.g., Broad, Feigl, Nagel, Jackson), or advocates of the so called *explanatory gap* postulate (coined by Levine), claim that science should provide direct knowledge of other subjects’ experiences, it may be assumed that their arguments involve a *category error* (Pigliucci, 2013). Indeed, the *hard problem*, formulated this way, “does not require a solution, but rather, a cure” (see Edelman et al., 2011, p. 5).

## Empirically Valid Questions

There are two standard questions that may be asked about subjective experience: the ‘why’ and the ‘how’ questions. As an empirically valid question, the ‘why’ question (i.e., ‘Why is there subjective experience?’) asks about function. In a naturalistic scenario, it actually just concerns *biological function*: i.e., the evolutionary or adaptive reasons for subjectivity, or the advantages furnished by subjective experiences for the functioning of some animal. Meanwhile, two slightly different ‘how’ questions (in the sense of ‘How does it arise?’) may be distinguished: one about the biological origins of subjectivity (‘How did subjectivity develop among species?’), the other about the generation of subjective states in a given organism (‘How are they produced?’). Both are, in fact, mechanistic questions, as the former asks about global or *developmental mechanisms*, the latter about local or *production mechanisms*.

## Consciousness, Information, and Subjectivity

### All Conscious States are Informational States

This claim is quite common nowadays; some would even say that “all the components of consciousness are solely information in various forms” (see Earl, 2014, pp. 8–9). Obviously the very notion of information is crucial here, as not all of its versions seem to fit with consciousness studies (Pockett, 2014). The currently most popular informational approach to consciousness, *integrated information theory* or IIT, began with a rather classical understanding of information (in terms of *uncertainty reduction*; see Tononi, 2004, 2008, p. 217). Nevertheless, Koch and Tononi claim that “IIT introduces a novel, non-Shannonian notion of information – integrated information – which can be measured as ‘differences that make a difference’ to a system from its intrinsic perspective, not relative to an observer” (Koch and Tononi, 2013). What notion of information is or should be applied here is a fascinating question in its own right. However, if being *internally differentiated* is sufficient for being informational for given system, as IIT assumes, then, indeed, all conscious states would seem to be informational states – since it is rather obvious that in order to be identified at all, states of consciousness must differ relative to one another when viewed from the internal (i.e., subjective) perspective of a given system.

### Not All Informational States are Conscious

That not all information processing is conscious is something that seems fairly obvious, and is apparently widely endorsed in cognitive science^2^. Some claim that not only lower-level, but also higher-level information processing, which engages *prefrontal areas*, is not necessarily conscious (van Gaal and Lamme, 2012) – not until *recurrent processing* enters in (e.g., Lamy et al., 2008). Others maintain that even executive, top-down control might be carried out unconsciously (Kiefer, 2012). What is significant for IIT is that even integrated informational states need not always reach consciousness – as Mudrik and other researchers, including Koch himself, have argued (Mudrik et al., 2011, 2014). In at least four cases, that might be the case: when the time and space of exposure is limited (*narrow spatiotemporal window*), when the information is insufficiently semantically complex, when it is not multimodal, and when it is not novel for the subject (see Mudrik et al., 2014, pp. 491–494). If this is so, then not all informational states available within a given system will necessarily be conscious, even if they are integrated.

### All Informational States are Subjective

This claim is by no means obvious. However, IIT does assume the *intrinsic existence* of all conscious – i.e., integrated – informational states (as an axiomatic, *Cartesian*, phenomenological feature; see Tononi and Koch, 2015, p. 5)^3^. Philosophically speaking, such an assumption is rather fundamental, as it is in some sense really positing a subjective ontology of consciousness (Searle, 1992, 2000a,b). A plausible, naturalistic justification of the subjectivity of informational states will be proposed below, yet without drawing any ontological consequences (as these are by no means definite).

The phenomenon of consciousness is known only *in situ* and *in vivo*. Hence, if it is to be described in informational terms, consideration should be given to what kind of information we are dealing with here, in the case of biological systems. To the extent that we understand the natural world, accessibility of information may be considered deeply embedded in our evolutionary background (Feinberg and Mallatt, 2013). Each and every creature is morphologically and physiologically attuned and sensitive only to specific sorts of informational resource (i.e., those most efficacious for its ancestors). In other words, *earthly creatures* (see Millikan, 2004, p. 9) are naturally constrained to making use of just certain wavelengths and frequencies of light and sound, certain chemicals, and only particular capacities for movement, sensation, etc. These phylogenetic limitations are further modified epigenetically and socially, becoming still more specific and unique at the phenotypical level (Fraga et al., 2005; Swaddle et al., 2005; Bossdorf et al., 2008; Ballestar, 2010; Migicovsky and Kovalchuk, 2011). Finally, informational states accessible within an individual organism, already heavily preselected, are shaped by its very own developmental history, since biological systems are structured by all their past and future encounters (especially connection patterns in their brains; see Frith, 2011). The ultimate form and content of states accessed by an individual subject will be determined by the subject’s current state and *situatedness* – that is, by the specific internal and external conditions the creature is actually subject to (e.g., individual physiological parameters, location in a particular space, contextual relations, ongoing engagements, expectations, etc.,). As a result, “(a)t any given moment, a process of integration of collective neuronal activity generates an interwoven pattern of responses unique to a particular animal at that particular moment of time” (Edelman et al., 2011, p. 3).

In conclusion, it may be said that information possessing biological value is always *individuated* (Jonkisz, 2015)^4^. And this may even be put strongly: all informational states, no matter whether conscious or integrated, are *biologically individuated* within a given organism – i.e., directly accessible only from its internal perspective, meaning only subjectively.

## Some Provisional Answers

All the factors described above contribute to the complex process of *biological individuation*, causing *individual differences* to occur in each and every organism at virtually every level (be it genetic, epigenetic, anatomical, behavioral etc.,). Even *monozygotic twins* reared together, or *clones*, differ significantly, as it is not possible to mimic all the environmental interactions and physiological conditions involved in forming each phenotype (Pfefferbaum et al., 2004; Fraga et al., 2005; Ballestar, 2010; Maiti et al., 2011; Freund et al., 2013). In the light of this, the conclusion that individual differences are also present at a given organism’s cognitive or epistemic level seems quite plausible (in the sense that what the organism is able to perceive or experience will also be unique, meaning directly accessible only from its perspective, i.e., subjectively). Ultimately, it may be said that the process of biological individuation furnishes, at the very least, a provisional answer to the question concerning the origins or developmental mechanisms of subjectivity (‘How did subjectivity develop among species?’). Below, a plausible hypothesis is presented which also answers the question concerning *biological function* (‘Why is there subjective experience?’).

What adaptive advantages might subjectivity furnish for the functioning of an animal? It is a rather obvious empirical observation, that the survival of an animal is determined by its ability to pick out the most efficient patterns of action and adapt them to the changing conditions of the moment. However, in a complex, ever-changing environment, organisms are potentially able to discern an infinite amount of information and an infinite number of states. Therefore, there is likely to be strong evolutionary pressure on their ability to minimize the more redundant states and select the most efficient ones. That is probably part of the reason why the plethora of possible informational resources is subject to such a degree of phylogenetic reduction and ontogenetic preselection (as described in “All Informational States are Subjective”). What remains accessible is what is potentially most useful, in a Bayesian sense, and hence most valuable biologically – both for a given species and for the individual organism. More specifically, one may say that those networks of experiential/informational associations that have worked well for an organism in the past form biases triggered by similar future occurrences. Attentional priorities for spotting differences (novelties) in the ongoing unfolding of informational states, together with the emotional markers ascribed to them, enable further learning and adaptation (*brain plasticity* and neuronal group selection, or so called *neural Darwinism*, account well for this picture; see Cleeremans, 2011; Edelman et al., 2011; Frith, 2011; Baars, 2012). From this perspective, organisms may be viewed as Bayesian systems specializing in anticipating what the future will bring and discriminating what is best for them from their individual, unique and private point of view – i.e., a subjective one. The process of formation of such an *individuated informational space* (accessed only subjectively), occurring in several distinct stages (described above), is likely to be the most efficient way of selecting biologically valuable information, and is therefore justified as far as the functioning of the animal is concerned. We may thus conclude that the hypothesis according to which subjectivity might be seen (functionally) as an *adaptive response to informational overflow* seems valid and plausible.

The hypothesized function of subjectivity (the selection of overflowing information) is partly supported by empirical evidence pertaining to both the bottom-up and the top-down influence of bodily factors on information processing (see Theeuwes, 2010; Fleming et al., 2010; Rochat, 2011; Shimono et al., 2012; Zhou et al., 2013; Pfeifer et al., 2014). The way our bodies are shaped (by their evolutionary background, developmental conditions, current engagements and actual physiological state) determine informational states’ availability, resulting in the formation of *individuated informational spaces* for each and every organism (the statistically most useful ones).

The foregoing considerations have introduced provisional answers for questions about the function of subjectivity (the ‘why’ question) and its developmental mechanisms (a global ‘how’ question). What still remains is the local ‘how’ question concerning the production of subjective experiences within an organism. The task of discovering experimentally the mechanisms responsible for generating states of consciousness has proved to be by far the most challenging one. We still seem far away from any sort of end to that journey, even though much knowledge about the neuronal areas engaged and the processes involved has been gathered (Metzinger, 2000; Crick and Koch, 2003; Hohwy, 2009; Lamme, 2010; Panagiotaropoulos et al., 2012; Baars, 2012). Amongst the findings so far, a few promising models have been elaborated: e.g., GWT and GNW, RP, and the more abstract IIT model (Baars, 1994, 1996; Dehaene et al., 1998; Dehaene and Naccache, 2001; Tononi, 2004, 2008; Lamme, 2006; Dehaene and Changeux, 2011; Baars et al., 2013; Tononi and Koch, 2015). Indeed, the *generation problem* seems to represent the most difficult challenge for science, though it does seem to remain tractable from the standpoint of an empirical science of consciousness. Undeniable proof that the hard problem had been solved would probably need to take the form of an at least minimally conscious artifact (Kurzweil, 2012; O’Regan, 2012). However, still more effective treatments, enabling a regaining of consciousness in patients who have lost vital components of the latter, would offer important indicators of the right path having been followed (Giacino, 2005).

## Conclusion

The article presents a provisional account of both the developmental mechanism of subjectivity (described here as *biological individuation*) and its function (described as an adaptive response to *informational overflow*). It also points to some fairly unconventional conclusions about the relation between subjectivity and consciousness. All conscious states count as informational (see “All Conscious States are Informational States”), but not all informational states (even those that are integrated) count as conscious (see “Not All Informational States are Conscious”). It is also argued that all informational states are subjective (biologically individuated, see “All Informational States are Subjective”). While it is generally considered quite acceptable to claim that all conscious states are subjective (as is entailed by “All Conscious States are Informational States” and “All Informational States are Subjective”), the conclusion that some subjective states are not conscious (as is entailed by “Not All Informational States are Conscious” and “All Informational States are Subjective”) remains rather unpopular.

Subjectivity, as an adaptive strategy for selecting the most useful informational resources, might in fact be dissociated from consciousness, at least functionally. (That idea was first developed in Jonkisz, 2009; the separation presented here have consequences whose significance is above all explanatory). Whereas subjectivity, as described here, primarily facilitates selection (based on Bayesian statistics), consciousness may be seen as a phenomenon that primarily facilitates action (Jonkisz, 2015). The overall picture may be briefly described as follows: both the bottom-up bodily factors (genetic, epigenetic, and developmental) and the top-down priming effects or expectations (based on prior experiences) serve not only to reduce the number of possible states, but also to bias accessible ones toward or against certain other informational states (Fleming et al., 2010; Theeuwes, 2010; Rochat, 2011; Shimono et al., 2012; Zhou et al., 2013; Pfeifer et al., 2014). Hence, every creature ultimately ‘works on’ a biologically individuated informational space that consists of all of the possible experiential/informational contents formed within the system^5^. Those of the subjective, informational contents that are actually utilized in action become conscious, where this is likely to occur in a gradational manner (see Jonkisz, 2015; for the crucial role of action, see also, e.g., Gibson, 1977, 1979; Noë, 2006; Engel et al., 2013; etc.,).

Our general conclusion, then, is that subjectivity, which develops through many stages of biological individuation, is inherited in the form of our *being-in-the-world*. We are all structurally, functionally, and phenomenally different (as already mentioned, even *monozygotic twins* and *clones*, similar as they appear to be and actually are, differ significantly, see Pfefferbaum et al., 2004; Fraga et al., 2005; Ballestar, 2010; Maiti et al., 2011; Freund et al., 2013). A complex and dynamic ecosystem, unique in itself, contains myriads of unique, individuated systems with their own private perspectives. Admittedly, subjective Bayesian systems are fallible in their heuristic processing, being prone to illusion and undetected error (Lotto and Purves, 2002; Rossano, 2003; Lotto, 2004), yet this is forgivable, as biological individuation has itself most likely brought about not only a balance between efficacy and economy in their interactions with their environment, but also the emergence of selves (Rochat, 2011).

## Author Contributions

The author confirms being the sole contributor of this work and approved it for publication.

## Conflict of Interest Statement

The author declares that the research was conducted in the absence of any commercial or financial relationships that could be construed as a potential conflict of interest.

## Footnotes

1 Subjectivity is understood in the article as the epistemic property of being privately or first-personally accessible (see also Jonkisz, 2012, pp. 60–61).

2 There are, however, exceptions to this claim: e.g., Chalmers, who claims that “…wherever there is a causal interaction, there is information, and wherever there is information, there is experience,” and that “Experience is information from the inside; physics information from the outside” (by ‘experience’ he means ‘phenomenal consciousness’; see Chalmers, 1996, p. 297 and p. 305).

3 IIT also postulates that a physical mechanism needs to exercise a “cause-effect power upon itself” in order to support this feature (pp. 7–8), and characterizes conscious experience as “maximally irreducible intrinsically conceptual structure” (or “Φmax”).

4 The notion of individuation has played a central role in the philosophical theories developed by Simondon, and fits neatly into the context being described in the article (Simondon, 2005; Iliadis, 2013).

5 Such an *individuated informational space* may be seen as analogous to the *Global Workspace* (Baars, 1996; Edelman et al., 2011; Baars et al., 2013): as an embodied, and probably extended or externalized, version of the latter.
